# TSC1/2 Signaling Complex Is Essential for Peripheral Naïve CD8^+^ T Cell Survival and Homeostasis in Mice

**DOI:** 10.1371/journal.pone.0030592

**Published:** 2012-02-21

**Authors:** Lianjun Zhang, Hongbing Zhang, Lanlan Li, Yi Xiao, Enyu Rao, Zhuang Miao, Hui Chen, Lina Sun, Hongran Li, Guangwei Liu, Yong Zhao

**Affiliations:** 1 Transplantation Biology Research Division, State Key Laboratory of Biomembrane and Membrane Biotechnology, Institute of Zoology, Chinese Academy of Sciences, Beijing, China; 2 Graduate University of the Chinese Academy of Sciences, Beijing, China; 3 Department of Physiology and Pathophysiology, National Laboratory of Medical Molecular Biology, Institute of Basic Medical Sciences and School of Basic Medicine, Chinese Academy of Medical Sciences and Peking Union Medical College, Beijing, China; French National Centre for Scientific Research, France

## Abstract

The PI3K-Akt-mTOR pathway plays crucial roles in regulating both innate and adaptive immunity. However, the role of TSC1, a critical negative regulator of mTOR, in peripheral T cell homeostasis remains elusive. With T cell-specific Tsc1 conditional knockout (Tsc1 KO) mice, we found that peripheral naïve CD8^+^ T cells but not CD4^+^ T cells were severely reduced. Tsc1 KO naïve CD8^+^ T cells showed profound survival defect in an adoptive transfer model and in culture with either stimulation of IL-7 or IL-15, despite comparable CD122 and CD127 expression between control and KO CD8^+^ T cells. IL-7 stimulated phosphorylation of Akt(S473) was diminished in Tsc1 KO naïve CD8^+^T cells due to hyperactive mTOR-mediated feedback suppression on PI3K-AKT signaling. Furthermore, impaired Foxo1/Foxo3a phosphorylation and increased pro-apoptotic Bim expression in Tsc1 KO naïve CD8^+^T cells were observed upon stimulation of IL-7. Collectively, our study suggests that TSC1 plays an essential role in regulating peripheral naïve CD8^+^ T cell homeostasis, possible via an mTOR-Akt-FoxO-Bim signaling pathway.

## Introduction

PI3K-Akt-mTOR signaling pathway plays crucial roles in regulating both innate and adaptive immunity [1]–[3]. In mammalian cells, mTOR can form two complexes which are called mTOR complex1(mTORC1) and mTOR complex2(mTORC2), respectively, via binding with different partner proteins. mTORC1 activity is negatively regulated by a heterodimeric complex composed of TSC1 (hamartin) and TSC2 (tuberin). The TSC1/2–mTOR pathway serves as a central regulator of cellular metabolism, survival, growth and differentiation through integrating various environmental cues [4]–[7]. TSC1/2-mTOR signaling pathway regulates the innate inflammatory response of macrophages and plasmacytoid dendritic cells in mice [1], [8]–[10]. Increasing evidence suggests that TSC1/2-mTOR pathway regulates T cell survival, anergy, trafficking, as well as the generation of different T cell subset differentiation [11]–[20]. However, the detailed role of TSC1/2 complex in naïve T cell survival and homeostasis remains to be studied.

In the present study, we generated the T cell-specific Tsc1 knockout mice by crossing Tsc1^loxp/loxp^ mice with transgenic mice that carried Lck proximal promoter-mediated Cre recombinase. We found that mTORC1 activity was significantly increased in Tsc1 null T cells, CD8^+^ but not CD4^+^T cells decreased dramatically in secondary lymphoid organs including spleen and lymph nodes (LNs) but not in the central lymph organ thymus. Upon transferring into syngeneic Rag1^−/−^ or irradiated immunocompetent recipients, Tsc1 KO naïve CD8^+^ T cells displayed apparent survival and homeostatic defects. Furthermore, Tsc1 KO naïve CD8^+^ T cells showed profound survival defects in cell culture with either IL-7 or IL-15, despite their comparable surface CD122 and CD127 expression and slightly decreased STAT5 phosphorylation in comparison with WT cells. However, phosphorylation of Akt(S473) in response to IL-7 stimulation was compromised in Tsc1 KO naïve CD8^+^T cells. Collectively, these data suggest that TSC1 is a critical regulator of naïve CD8^+^ T cell survival and homeostasis *in vivo*.

## Results

### TSC1/2 is preferentially expressed in CD8^+^T cells

**Figure 1:**
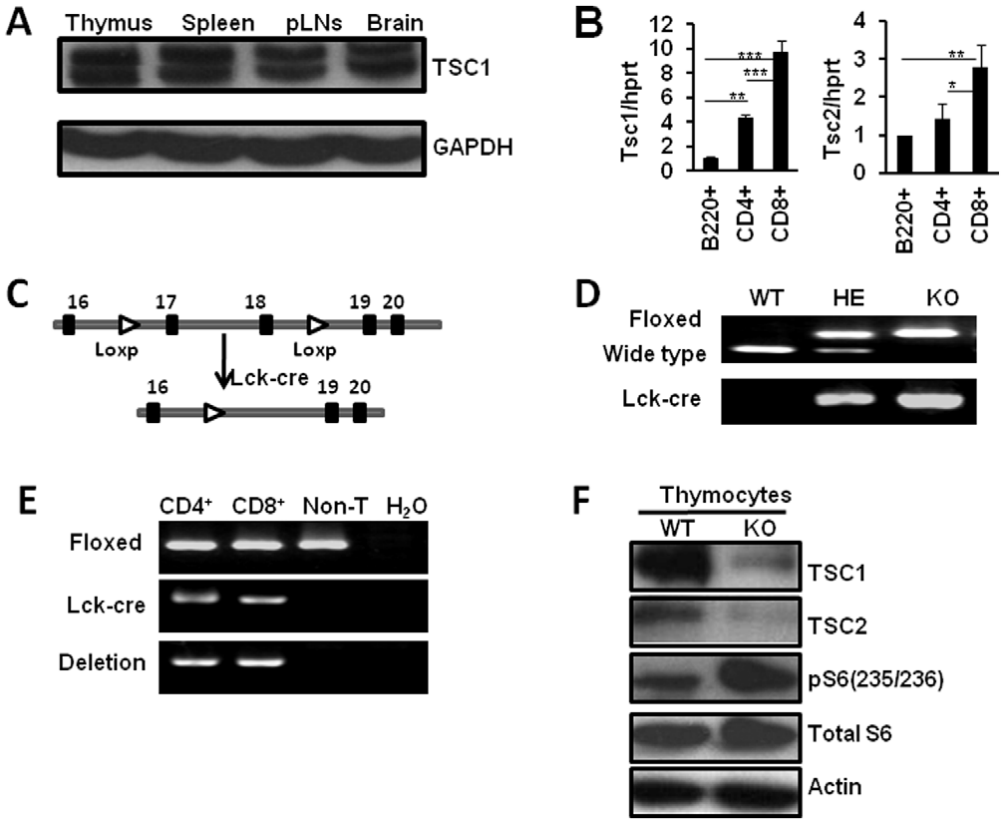
Generation of the T cell-specific Tsc1 KO mice. (**A**) Western blot analysis showed TSC1 was expressed in thymus, spleen as well as pLNs of WT B6 mice, brain serves as the positive control. (**B**) Tsc1 and Tsc2 expression level in splenic CD8^+^T cells was higher than that in B cells and CD4^+^T cells as detected by Real-time PCR analysis. *p<0.05; **p<0.01; ***p<0.001 compared with the indicated groups. Date were shown as Mean±SD (N = 6). (**C**) Schematic representation of deletion of Tsc1 exons 17 and 18 by Lck-Cre-mediated recombination in T cells. (**D**) Genotyping of wild type (Tsc1^+/+^, WT), heterozygous(Lck-cre^+^Tsc1^loxp/+^, HE) and knockout (Lck-cre^+^Tsc1^loxp/loxp^, KO) mice with tail genomic DNA. (**E**) Detection of Tsc1 deletion in purified CD8^+^T cells, CD4^+^T cells and non-T cells from spleen of Tsc1 KO mice. Tsc1 was specifically deleted in T cells. (**F**) Western blot analysis showed efficient deletion of TSC1 and TSC2 protein expression in thymocytes. Phosphorylated S6 level was significantly increased in Tsc1 KO mice.

As a first step in characterizing the physiological function of TSC1 in mouse T cells, we examined the expression patterns of Tsc1 in thymus and peripheral lymphoid organs. By Western blot, we found that TSC1 was expressed in thymus, spleen as well as peripheral lymph nodes (pLNs), brain served as the positive control as it has been described to highly express Tsc1/2 (Fig. 1A). We also employed real-time PCR assay to check the expression patterns of Tsc1 and Tsc2 in peripheral T cell subsets. Compared with B cells and CD4^+^ T cells, splenic CD8^+^ T cells showed higher expression of Tsc1 and Tsc2 (Fig. 1B). These observations suggest a potential role for TSC1/2 in T cells, especially in CD8^+^ T cells.

### Naïve CD8^+^ T cells, not CD4^+^T cells, were significantly decreased in the periphery of TSC1 KO mice

To study how TSC1/2 regulates the T cell development and function, we generated mice with specific deletion of Tsc1 in T cells by crossing Tsc1^loxp/loxp^ mice with transgenic mice that carried Lck proximal promoter-mediated Cre recombinase. After several rounds of crossing, we obtained mice with Tsc1 homozygous deletion in T cells (Lck-cre^+^Tsc1^loxp/loxp^; Tsc1 KO), Tsc1 heterozygous deletion in T cells (Lck-cre^+^ Tsc1^loxp/+^) and wild type Tsc1 in T cells (Tsc1^loxp/loxp^). Deletion of Tsc1 exons 17 and 18 by Lck-Cre-mediated recombination specifically in peripheral CD4^+^ and CD8^+^T cells but not in B cells was detected (Fig. 1C–E). By Western blot, we found TSC1 expression was almost absent in thymocytes of Lck-cre^+^Tsc1^loxp/loxp^(Tsc1 KO) mice, consequently TSC2 expression was also diminished as TSC1 was previously described to stabilize TSC2 [21], [22], indicating the efficient removal of TSC1/2 specifically in T cells (Fig. 1F). Consistently, we observed the increased level of S6 phosphorylation in Tsc1 KO thymocytes, which demonstrated that mTORC1 activity was significantly increased in the absence of TSC1 in T cells (Fig. 1F). S6 phosphorylation was also increased in peripheral CD4^+^ and CD8^+^ T cells of Tsc1 KO mice and was suppressed by rapamycin (data not shown).

**Figure 2:**
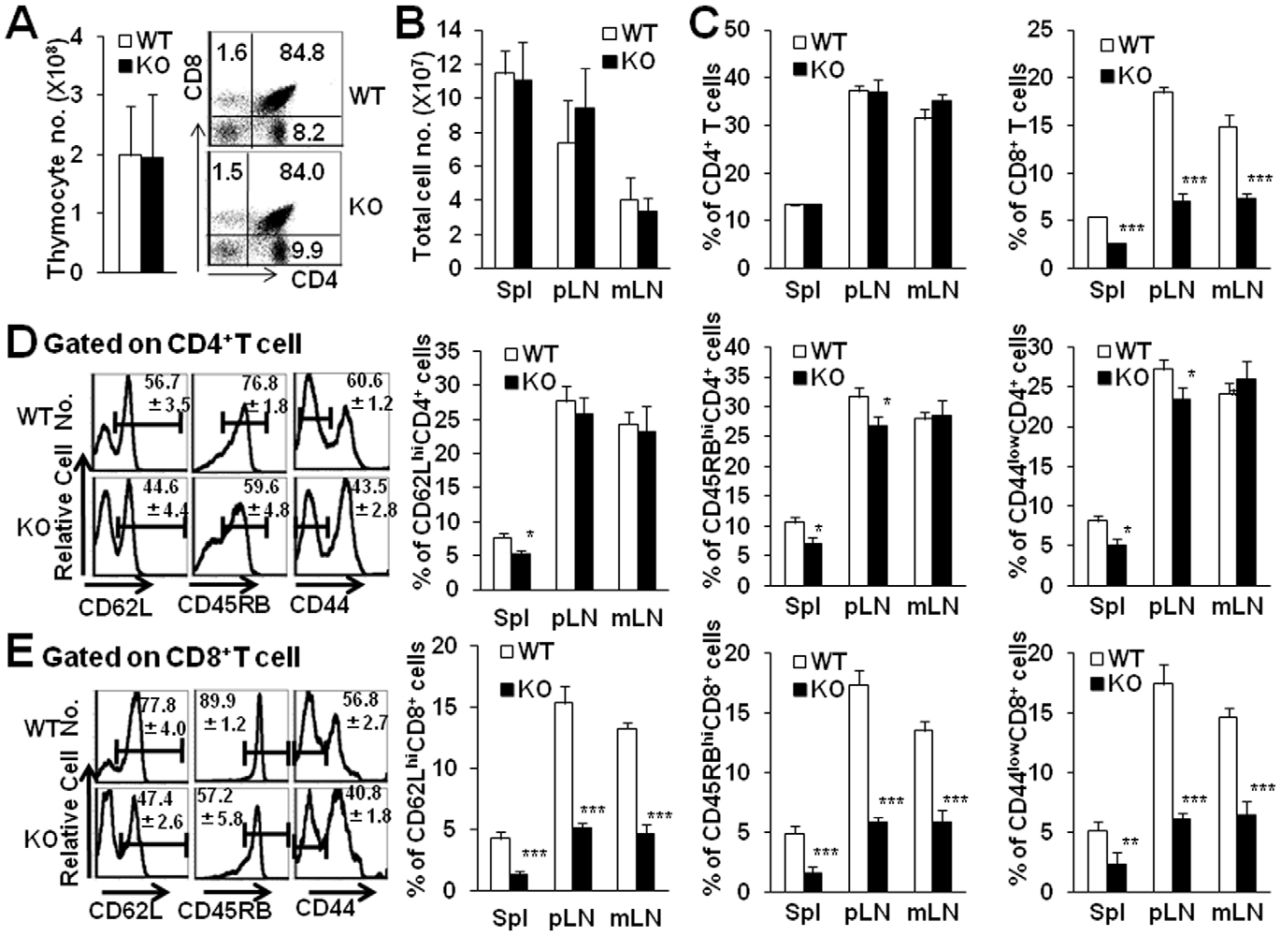
Naïve CD8^+^ but not CD4^+^ T cells were significantly decreased in the periphery of Tsc1 KO mice. The peripheral T cell subsets of WT and Tsc1 KO mice were detected using FCM 4–5 wks after birth. **A.** Total thymic cell number and thymocyte subset profile of Tsc1 KO mice were identical as WT littermates. **B.** Total cell number of spleen, pLNs and mLNs of WT and Tsc1 KO mice. **C.** The percentage of CD8^+^T but not CD4^+^T cells decreased dramatically in all peripheral lymphoid organs of Tsc1 KO mice. **D.** The FACS profile analysis of CD62L, CD45RB and CD44 on pLN CD4^+^ T cells was evaluated. The frequency of naïve CD62L^hi^, CD45RB^hi^ and CD44^low^ CD4^+^T cells in spleen, pLN and mLN of Tsc1 KO mice was summarized. **E.** The FACS profile analysis of CD62L, CD45RB and CD44 on the gated pLN CD8^+^ T cells was evaluated. The frequency of naïve CD62L^hi^, CD45RB^hi^ and CD44^low^ CD8^+^T cells in spleen, pLN and mLN of Tsc1 KO mice was summarized. *p<0.05; **p<0.01; ***p<0.001. Data were shown as Mean±SD (N = 6).

We next determined whether Tsc1/2 deficiency affected the composition of T cell compartments in peripheral and central lymphoid organs. As shown in Fig. 2, the total thymic cell number, and the percentage and cell number of CD4 or CD8 single positive cells in the thymus of Tsc1 KO mice were identical as WT littermates (Fig. 2A). No significant alteration for the cellularity in peripheral organs including spleen, pLNs and mesenteric lymphoid nodes (mLNs) in Tsc1 KO mice compared with age- and sex-matched WT littermate. However, the percentage of CD8^+^T cells decreased dramatically in all peripheral lymphoid organs of Tsc1 KO mice (Fig. 2B and C, P<0.001), whereas the frequency of CD4^+^T cells was comparable between Tsc1 KO mice and control littermates. The decreased level of CD8^+^T cells in peripheral lymphoid organs was not due to the enhanced migration of CD8^+^T cells into liver and lung, as we found the percentages and total cell number of CD8^+^T cells in the livers of WT or Tsc1 KO mice were comparable (Figure S1). Consistently, the frequency of B220^+^ cells was increased in pLNs and mLNs of Tsc1 KO mice. The absolute number of CD8^+^ but not CD4^+^T cells was significantly decreased in these organs of Tsc1 KO mice (data not shown). Thus, the ratio of CD4^+^ to CD8^+^T cells increased significantly in pLNs of Tsc1 KO mice as well (5.25±0.47 compared with 2.03±0.25). The increased ratios of CD4^+^ to CD8^+^T cells were also observed in spleens as well as mLNs of Tsc1 KO mice.

To further determine the phenotype or activation status of T cells in Tsc1 KO mice, the expression of naïve, effector/memory surface markers including CD62L, CD45RB and CD44 on either CD4^+^ or CD8^+^T cells was evaluated using FCM. Though the frequency of naïve CD62L^hi^, CD45RB^hi^ and CD44^low^ CD4^+^T cells in spleen, pLNs and mLNs of Tsc1 KO mice was kept normal as WT mice (Fig. 2D), remarkably decreased percentages of naïve CD62L^hi^, CD45RB^hi^ and CD44^low^ CD8^+^T cells in spleen, pLNs and mLNs of Tsc1 KO mice were observed (Fig. 2E, P<0.001). The reduction of peripheral naïve CD8^+^T cells in Tsc1 KO mice was not caused by Cre-induced toxicity, as Lck-cre+, Tsc1^loxp/+^mice showed normal levels of peripheral naïve CD8^+^T cells. The different expression intensity of TSC1/2 in CD4^+^ and CD8^+^T cells and the distinct alteration of CD4^+^ and CD8^+^T cells in the periphery of Tsc1 KO mice collectively indicates that TSC1/2 may play differential roles in regulating T cell subsets in mice.

### TSC1 is required for naive CD8^+^ T cell survival and homeostasis *in vivo*

Based on our data mentioned above, we speculated that peripheral survival deficiency of naïve CD8^+^T cells might exist in Tsc1 KO mice. To test this hypothesis, we employed mouse models with an adoptive transfer of sorted syngeneic naïve CD8^+^T cells as well as the *in vitro* IL-7 and IL-15-dependent naïve CD8^+^T cell survival assays to address the effect of TSC1/2 on the survival capacity of peripheral naïve CD8^+^T cells.

**Figure 3:**
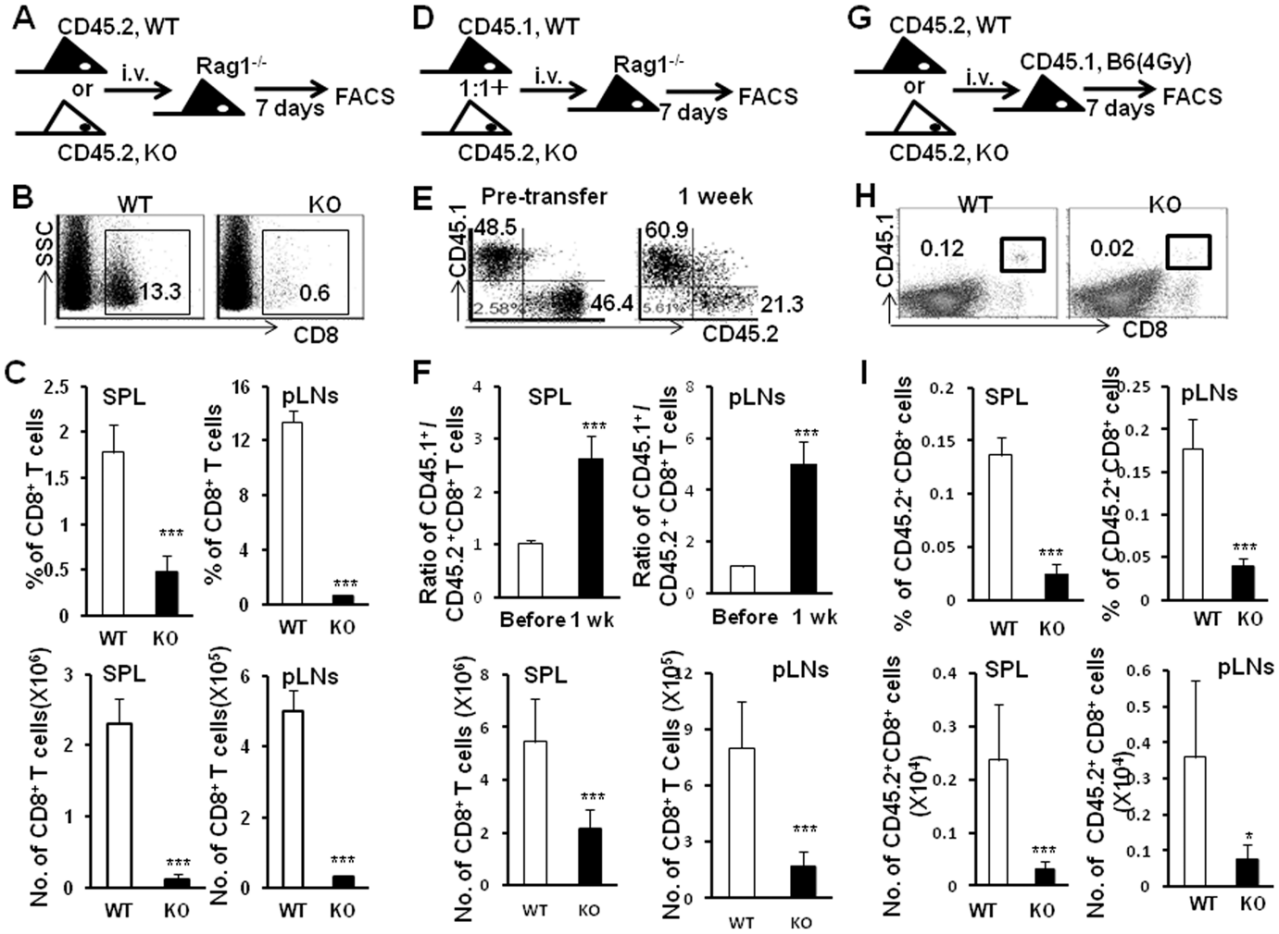
TSC1 is required for naive CD8^+^ T cell homeostasis in adoptive transfer mouse models. Sorted naïve CD8^+^T cells were adoptively transferred into syngeneic Rag1^−/−^ or irradiated recipients. The levels of transferred naïve CD8^+^T cells in spleen and pLNs of recipients were determined 7 days after transfer. (**A**) Schematic representation of adoptive transfer of either WT or Tsc1 KO CD45.2^+^ naïve CD8^+^ T cells into Rag^−/−^ host. (**B**) One representative staining of CD8^+^T cells in pLN of Rag1^−/−^ mice after transfer of sorted naïve CD8^+^T cells. (**C**) Tsc1 KO CD45.2^+^ naïve CD8^+^ T cells showed homeostatic defect in spleen and pLNs. Statistic analysis of percentage and numbers of transferred CD8^+^T cells in spleen as well as pLN. (**D**) Schematic representation of adoptive transfer of mixed(1∶1) population of WT CD45.1^+^ and Tsc1 KO CD45.2^+^ naïve CD8^+^T cells into Rag^−/−^ host. (**E**) One representative staining of CD45.1 and CD45.2 on gated CD8^+^T cells in pLNs of Rag1^−/−^ mice 7 days after transfer of sorted naïve CD8^+^T cells. (**F**) The ratio between WT CD45.1^+^CD8^+^ and Tsc1 KO CD45.2^+^CD8^+^ T cells as well as the frequency of CD45.1^+^ or CD45.2^+^CD8^+^ T cells in spleen and pLNs were summarized. (**G**) Schematic representation of adoptive transfer of either WT or Tsc1 KO CD45.2^+^ naïve CD8^+^ T cells into sublethally irradiated(4 Gy) CD45.1^+^ host. (**H**) One representative staining of CD45.1 and CD8 for pLN cells of recipients 7 days after transfer of sorted naïve CD8^+^T cells. (**I**) Lower percentage and cell number of Tsc1 KO CD45.2^+^ naïve CD8^+^ T cells in spleen and pLNs. *p<0.05, **p<0.01, and ***p<0.001 compared with WT group. Data were shown as Mean±SD (3–5 mice each group). One representative of two or three independent experiments with identical results was shown.

Adoptive transfer mouse models were commonly employed in studies of peripheral T cell survival and homeostasis [23], [24]. By one week after adoptive transfer of sorted either CD45.2^+^ WT or CD45.2^+^Tsc1 KO naïve CD8^+^T cells into Rag^−/−^ syngeneic recipients (Fig. 3A), significantly lower percentages and cell number of Tsc1 KO naïve CD8^+^ T cells in spleens and pLNs of recipients were observed compared with WT naïve CD8^+^T cells (Fig. 3B and C, P<0.001). When both CD45.2^+^Tsc1 KO and CD45.1^+^WT naïve CD8^+^T cells at a ratio of 1∶1 were simultaneously transferred into Rag1^−/−^ mice (Fig. 3D), the ratio of WT to Tsc1KO naïve CD8^+^T cells increased to 2.6±0.4∶1 and 5.0±0.9∶1 in spleens and pLNs of recipients, respectively. The percentages and cell number of Tsc1 KO naïve CD8^+^T cells were significantly lower than those of WT naïve CD8^+^T cells in spleens and pLNs as well (Fig. 3E and F, P<0.001). As Rag1^−/−^ mice were T/B cell deficient and might drive extensive homeostatic proliferation of naive T cells due to lymphopenia [24], we therefore adoptively transferred CD45.2^+^Tsc1 KO or CD45.2^+^WT naïve CD8^+^T cells into 4Gy-irriadiated immunocompetent CD45.1^+^syngeneic C57BL/6 recipients (Fig. 3G). Consistent with the results in T cell-deficient recipients, significantly decreased percentage and cell number of CD45.2^+^Tsc1 KO CD8^+^T cells were detected in irradiated immunocompetent B6 recipient mice compared with CD45.2^+^WT naïve CD8^+^T cells (Fig. 3H and I, P<0.001). This was not due to proliferative deficiency as these CD45.2^+^Tsc1 KO CD8+ T cells incorporated comparable level of BrdU as that of CD45.2^+^WT naïve CD8^+^ T cells (data not shown). Moreover, it might not be caused by peripheral migration or trafficking defects of Tsc1 KO naïve CD8^+^T cells, because we found significantly decreased percentages of Tsc1 KO naïve CD8^+^T cells in all peripheral lymphoid tissues such as PBL, spleen, pLNs as well as mLNs (Fig. 3H and I). Taken together, these data suggest that TSC1 is a critical regulator of naïve CD8^+^ T cell survival and homeostasis *in vivo*.

### The poor response to IL-7 and IL-15 of Tsc1 KO naive CD8^+^ T cells *in vitro*

**Figure 4:**
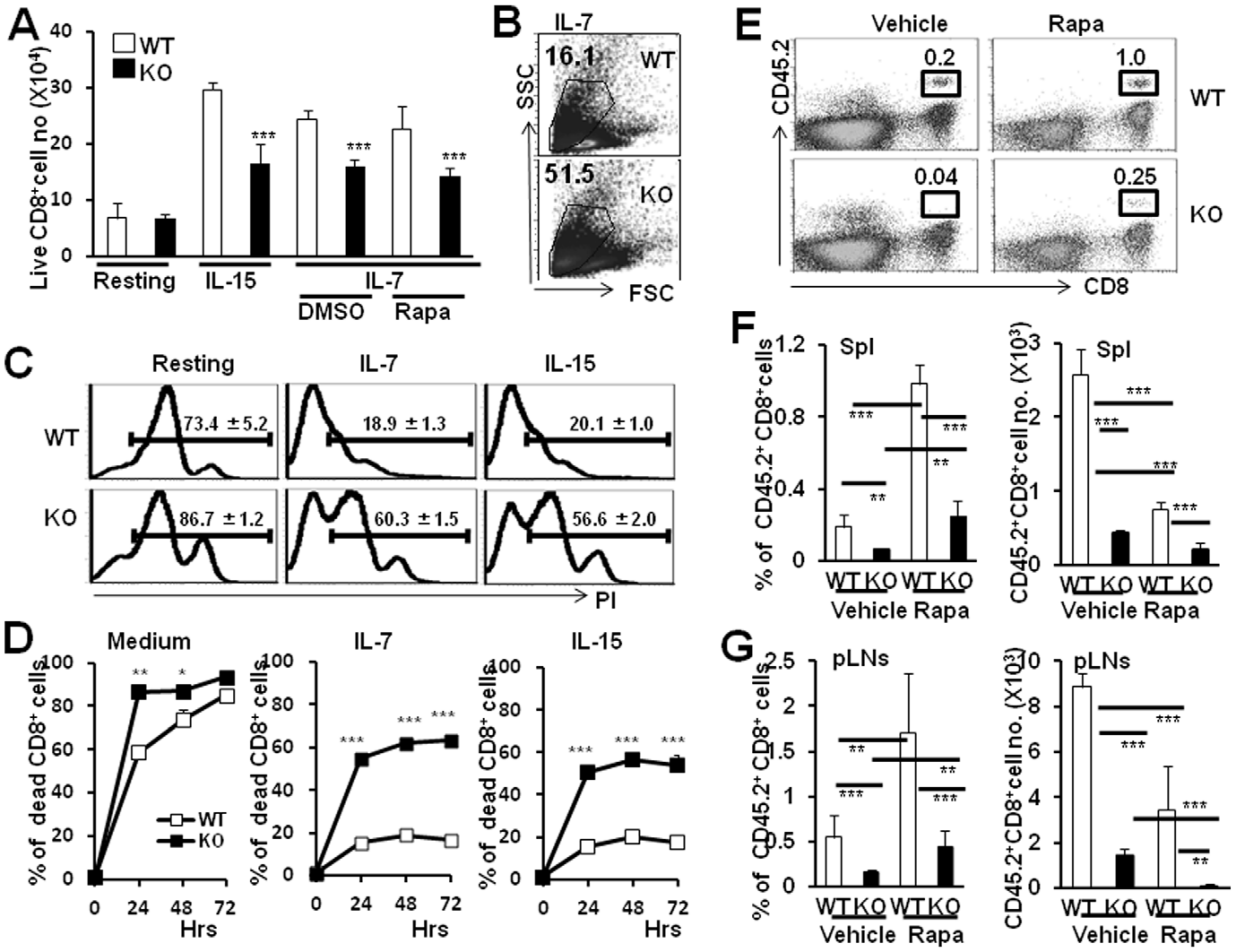
TSC1 regulates naïve CD8^+^ T cell survival to IL-7 or IL-15 in a rapamycin-insensitive manner. Sorted WT and Tsc1 KO mouse naïve CD8^+^CD44^low^ T cells were cultured in medium alone or supplemented with IL-15 or IL-7(in the presence or absence of Rapa) for 24 hrs or the below indicated time points. (**A**) Significantly decreased live cell number of Tsc1 KO naïve CD8^+^T cells than WT naïve CD8^+^T cells in the presence of IL-15 or IL-7 for 24 hours. Live cells were determined by trypan blue exclusion assay. (**B**) Tsc1 KO naïve CD8^+^ T cells showed severe atrophy in the presence of IL-7 for 24 hrs. (**C**) One representative of PI staining in gated CD8^+^T cells after culture with or without IL-7 and IL-15. (**D**) Percentage of cell death was measured by PI staining and statistically analyzed. The above data were one representative of three separate experiments, with three wells in each group. (**E**) Representative staining of CD45.2 and CD8 for pLN cells of CD45.1^+^ host in the presence or absence of Rapa 7 days after transfer. Either sorted WT or Tsc1 KO CD45.2^+^ naïve CD8^+^ T cells were adoptively transferred into sublethally irradiated(4 Gy) CD45.1^+^ host and the donor cells were determined by CD8 and CD45.2 staining. (**F**) Significantly decreased percentage and cell number of Tsc1 KO CD45.2^+^ naïve CD8^+^ T cells in spleen of sublethally irradiated (4 Gy) CD45.1^+^ host in the presence or absence of Rapa. (**G**) Significantly decreased percentage and cell number of Tsc1 KO CD45.2^+^ naïve CD8^+^ T cells in pLNs of sublethally irradiated (4 Gy) CD45.1^+^ host in the presence or absence of Rapa. Data were shown as Mean±SD (3 mice each group). *p<0.05; **p<0.01; ***p<0.001 compared with the indicated groups.

The peripheral T cell pool is constant throughout life and active maintenance of accurate peripheral naïve T cell pool depends on multiple homeostatic mechanisms [25]–[29]. Survival of naïve CD8^+^T cells is dependent on the presence of MHC I binding to peptide antigens [30]. In addition to TCR signals, cytokines such as IL-7 and IL-15 are critically required for the homeostatic survival of naïve T cells [23], [31]–[35], via the substantial induction of anti-apoptotic Bcl-2 expression or destabilization of p27^Kip1^ [36]–[38]. To determine whether Tsc1 KO naïve CD8^+^T cells were refractory to IL-7 and IL-15 mediated survival or not, we examined the Tsc1 KO naïve CD8^+^T cell survival ability in the presence or absence of IL-7 or IL-15 *in vitro*. As shown in Fig. 4, significantly increased survival frequency of WT naïve CD8^+^T cells by IL-15 or IL-7 was observed after culture for 24 hours. However, Tsc1 KO naïve CD8^+^ T cells displayed impaired survival upon stimulation with IL-7 or IL-15 for 24 hours (Fig. 4A, P<0.001). Consistently, naïve CD8^+^T cells isolated from Tsc1 KO mice exhibited significantly more cell death than WT cells by 24 hours in the presence of IL-7 and IL-15 *in vitro* as determined by cell atrophy (Fig. 4B) and PI staining (Fig. 4C and D).

It was reported that loss of TSC1 in cells resulted in mTOR hyper-activation [22], we thus added mTOR inhibitor, rapamycin (Rapa), into the naïve CD8^+^T cell culture system with IL-7. The survival defects of Tsc1KO naïve T cells in response to IL-7 was not rescued by Rapa treatment *in vitro* (Fig. 4A, P>0.05). Likewise, we treated CD45.2^+^ WT or Tsc1 KO naïve CD8^+^T cell-transferred CD45.1^+^ B6 mice with Rapa (i.p.) for 1 week, Rapa increased the percentage of both WT or Tsc1 KO naïve CD45.2^+^CD8^+^T cells in spleens and pLNs, possibly due to higher sensitivity to Rapa of other immune cells. However, significantly decreased cell number of WT and Tsc1 KO naïve CD45.2^+^CD8^+^T cells was observed in the presence of Rapa. Importantly, Rapa treatment failed to rescue the survival defect of Tsc1 KO naïve CD8^+^T cells *in vivo* (Fig. 4E–G, P>0.05). These data collectively indicate that TSC1/2 may regulate naïve CD8^+^T cell survival in Rapa-insensitive manner.

### Decreased Akt-FoxO1/FoxO3a phosphorylation level in Tsc1 KO naïve CD8^+^ T cells in response to IL-7 stimulation

**Figure 5:**
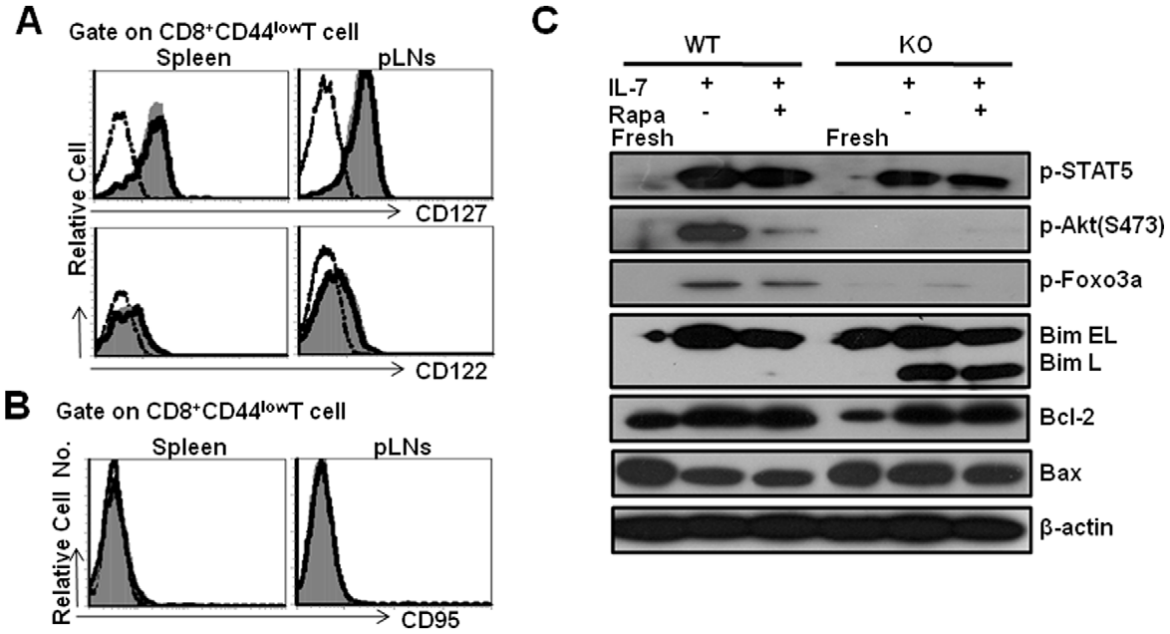
Decreased Akt-FoxO1/FoxO3a phosphorylation of Tsc1 KO naïve CD8^+^T cells in response to IL-7. The expression of CD122, CD127 and CD95 on naïve CD8^+^T cells was determined by gating CD8^+^CD44^low^ cells. Comparable surface CD122, CD127 (**A**) and CD95 (**B**) expression in Tsc1 KO naïve CD8^+^ T cells. The dash-dot line represents staining with an isotype control antibody. The histograms (gray) represent WT whereas the open histograms with solid line show Tsc1 KO staining pattern. Cells are gated with CD8^+^CD44^low^ population. Representative data are shown from one of two separate experiments, with three mice in each group. (**C**) Western blot analysis of mTORC2-Akt-FoxO1/FoxO3a-Bim axis in naïve CD8^+^ T cells after stimulation with IL-7. Sorted naïve CD8^+^T cells were cultured with IL-7 in the presence of Rapa or not for 24 hrs. The freshly isolated WT or Tsc1 KO naïve CD8^+^T cells were used as a control. One representative is shown from two or three separate experiments.

It was previously reported that CD127(IL-7Rα) expression is highly required for CD8^+^T cell survival and homeostatic proliferation. To investigate the potential reason for the survival defects of Tsc1 KO naïve CD8^+^T cells in response to IL-7 or IL-15, we first examined the expression levels of CD127, CD122(IL-15Rβ) or CD132(γc chain) in naïve CD8^+^T cells of WT or Tsc1 KO mice. However, cell surface CD127, CD122 or CD132 expression on Tsc1 KO naïve CD8^+^T cells was similar as WT CD8^+^T cells (Fig. 5A). Tsc1 KO naïve CD8^+^ T cells did not express increased levels of the death receptor Fas compared with WT naïve CD8^+^ T cells (Fig. 5B). IL-7 causes STAT5 phosphorylation via activation of Jak1 or Jak3. In this respect, IL-7 promotes cell survival by preventing the mitochondrial pathway of apoptosis, among which Bcl-2 is the predominant one [37], whereas other anti-apoptotic molecules such as Bcl-xl are barely expressed in naïve T cells. Upon IL-7 stimulation, we found slightly decreased STAT5 phosphorylation in Tsc1 KO naïve CD8^+^ T cells. Phosphorylation of Akt(S473) in Tsc1 KO naïve CD8^+^ T cells was not evident after IL-7 stimulation while IL-7 induced significant amount of Akt(S473) phosphorylation in WT naïve CD8^+^ T cells(Fig. 5C). It is reported that phosphorylation of Akt is important for FoxO1(Thr24) and FoxO3a(Thr32) phosphorylation which critically regulates FoxO1/FoxO3a transcriptional activity [39]. Once phosphorylated, FoxO1/FoxO3a is excluded from the nucleus and becomes transcriptionally inactive. In the present study, IL-7 treatment induced significantly higher level of FoxO1(Thr24) and FoxO3a(Thr32) phosphorylation in WT naïve CD8^+^T cells but not in Tsc1 KO naïve CD8^+^T cells.

It is well known that naïve T cell survival critically depends on the balanced expression of the pro-survival Bcl-2 and pro-apoptotic protein Bim [37]. Phosphorylated forms of FoxO3a directly inhibits the pro-apoptotic Bim expression to maintain central memory CD4^+^T cells(CD4^+^T_CM_) longevity and persistence in mice [40]. In IL-7 treated Tsc1 KO naïve CD8^+^T cells, Bim L expression was dramatically increased which was consistent with absence of Akt-mediated FoxO1(Thr24) and FoxO3a(Thr32) phosphorylation, while no Bim L expression was detected in IL-7-treated WT naïve CD8^+^T cells, even though both cells expressed similar levels of Bim EL(Fig. 5C). In addition, the Bcl-2 and Bax expression in Tsc1 KO naïve CD8^+^ T cells were identical with WT naïve CD8^+^T cells even in the presence of IL-7, excluding the possibility that the involvement of Bcl-2/Bax-related pathways in TSC1-mediated survival of naïve CD8^+^T cells.

## Discussion

**Figure 6:**
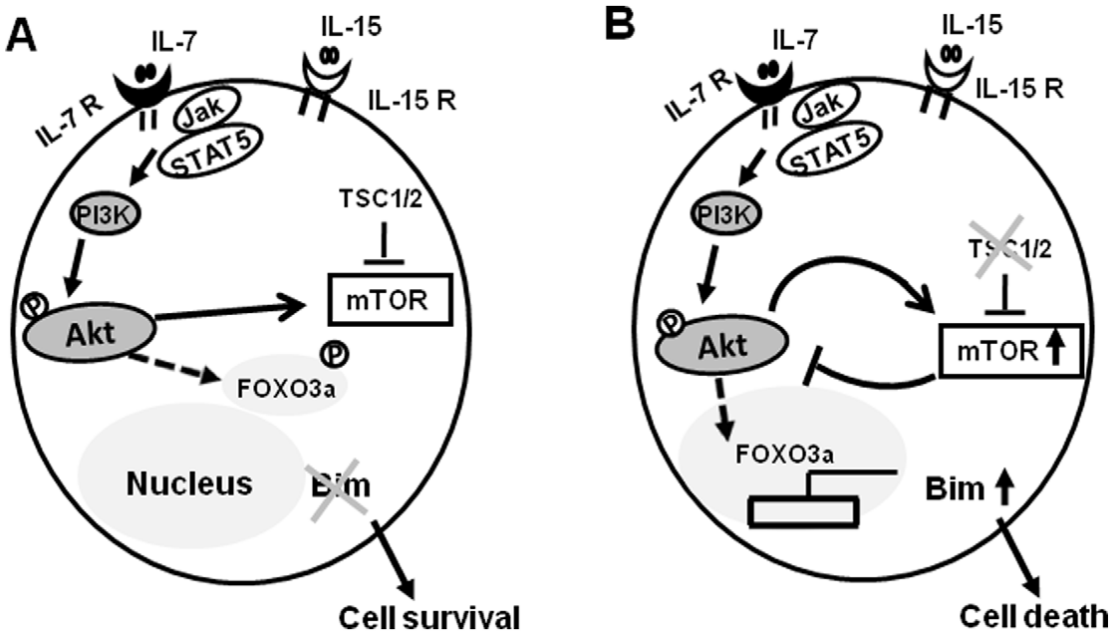
Schematic illustration of TSC1 in regulating naïve CD8^+^T cell survival in response to IL-7. (**A**) IL-7 stimulation triggers PI3K-Akt activation in naïve CD8^+^ T cells, which followed by FoxO1/FoxO3a phosphorylation. FoxO1/FoxO3a phosphorylation leads to their translocation into the cytoplasm from the nucleus, in this regard, naïve CD8^+^ T cell survival was maintained as the pro-apoptotic Bim expression was turned off. (**B**) Absence of TSC1 causes hyper-activation of mTOR which leads to suppression of PI3K-Akt signaling pathway. IL-7 stimulation of Akt activation is compromised in Tsc1 KO naïve T cells and thus Akt-dependent phosphorylation of FoxO1/FoxO3a is decreased. The accumulation of FoxO1/FoxO3a in the nucleus drives the pro-apoptotic Bim expression which promotes naïve CD8^+^ T cell death.

In the present study, using adoptive transfer mouse models, we clearly demonstrates that TSC1/2 signaling complex, an important negative regulator of mTOR, is essential for peripheral naïve CD8^+^T cell survival and homeostasis, which is consistent with recent studies(Fig. 6) [19], [20]. The defects of naïve CD8^+^T cell survival in the periphery of Tsc1 KO mice are largely intrinsic to mature peripheral naïve CD8^+^T cells, as evidenced by the poor survival of Tsc1 KO naive CD8^+^T cells when both WT and Tsc1 KO naïve CD8^+^T cells were co-transferred into synegeneic recipient mice. The normal distribution of thymocyte subsets and peripheral CD4^+^T cells in Tsc1 KO mice indicate that the distinct role of TSC1/2 signaling complex in peripheral naïve CD8^+^T cells at least in mice. The differential effects of TSC1/2 signaling complex on CD4^+^ and CD8^+^T cells are possibly related to the higher Tsc1/2 expression in CD8^+^T cells than CD4^+^T cells.

CD127 expression is required for naïve CD8^+^T cell survival and homeostatic proliferation. In Tsc1 KO naïve CD8^+^T cells, we failed to find significantly decreased expression of cell surface CD127, CD122 or CD132, suggesting that TSC1/2 signaling complex does not regulate IL-7 or IL-15 receptor expression on naïve CD8^+^T cells. In addition, Tsc1 KO naïve CD8^+^ T cells did not up-regulate Fas expression compared with WT naïve CD8^+^ T cells. Our data rule out the possibility that the poor survival of Tsc1 KO naïve CD8^+^T cells is due to the decreased cytokine receptor expression or increased cell death receptor expression. IL-7 initiates Jak1 or Jak3 activation which followed by STAT5 phosphorylation and promotes cell survival mainly through anti-apoptotic Bcl-2 up-regulation. Upon IL-7 stimulation, Bcl-2 up-regulation was not disrupted in Tsc1 KO naïve CD8^+^ T cells though STAT5 phosphorylation level was slightly decreased. Moreover, the pro-apoptotic Bax expression was similar between IL-7 stimulated Tsc1 KO naïve CD8^+^ T cells and WT cells, excluding the possibility that the involvement of Bcl-2/Bax associated pathways in TSC1-mediated survival of naïve CD8^+^T cells.

In our study, phosphorylation of Akt(S473) in Tsc1 KO naïve CD8^+^T cells was compromised after IL-7 stimulation while IL-7 induced a significant increase of Akt(S473) phosphorylation in WT naïve CD8^+^ T cells. Our study suggested that absence of Tsc1 caused a severe deficiency of IL-7 or IL-15-induced Akt activation in naïve CD8^+^ T cells. Ser473 phosphorylation is important for FoxO1(Thr24) and FoxO3a(Thr32) phosphorylation which plays an essential role in control of T cell homeostasis [24], [40], [41]. In WT naïve CD8^+^T cells, IL-7 induced significantly higher level of FoxO1(Thr24) and FoxO3a(Thr32) phosphorylation. However, IL-7 failed to induce FoxO1(Thr24) and FoxO3a(Thr32) phosphorylation in Tsc1 KO naïve CD8^+^T cells, which was consistent with the deficiency of Akt phosphorylation and activation in these cells. It was recently demonstrated that phosphorylated forms of FoxO3a could directly inhibit the pro-apoptotic Bim expression to maintain central memory CD4^+^T cells(CD4^+^T_CM_) longevity and persistence in mice [40]. In the present study, we found that Bim L expression was dramatically increased in IL-7 treated Tsc1 KO naïve CD8^+^T cells, while no Bim L expression was detected in IL-7-treated WT naïve CD8^+^T cells, even though both cells expressed similar levels of Bim EL.

Taken together, TSC1 critically regulates peripheral naïve CD8^+^T cell survival and homeostasis. Loss of TSC1 leads to mTOR-mediated inhibition of PI3K-AKT in naïve CD8^+^T cells. The TSC1/2-mTOR-Akt-FoxO-Bim anti-apoptosis pathway is essential in the maintenance of naïve CD8^+^T cell survival and homeostasis in mice.

## Materials and Methods

### Animals

Tsc1^loxp/loxp^ mice were generated by D.J. Kwiatkowski [42]. T cell specific Tsc1 conditional knockout mice were obtained by crossing Tsc1^loxp/loxp^ mice with transgenic mice that carried Lck proximal promoter-mediated Cre recombinase which had a C57BL/6J background (Kindly provided by Dr. Hua Han) [43]. After multiple rounds of crossing, we obtained homozygous mutant mice lacking Tsc1 in T cells (Lck-cre^+^Tsc1^loxp/loxp^) and heterozygous mice (Lck-cre^+^Tsc1^loxp/+^) mainly in C57BL/6 backgrounds. Both Lck-Cre-, Tsc1^loxp/loxp^ and Lck-cre^+^Tsc1^loxp/+^ mice served as control. Rag1^−/−^ mice were obtained from Center of Model Animal Research at Nanjing University, China. All animals were maintained in microisolator cages in a specific-pathogen free facility. All experiments were approved by the Animal Ethics Committee of the Institute of Zoology (IOZ2008012).

### mAbs and chemical reagents

The following mAbs were purchased from BD Biosciences PharMingen (San Diego, CA): anti-mCD4-FITC, anti-mTCRβ-PE, anti-mCD8α-PE, anti-mCD44-PE, anti-mCD45RB-PE, anti-mCD62L-PE, anti-mCD95-FITC, anti-mCD122-PE, anti-mCD127-PE, anti-mCD45.1-PE and anti-mCD45.2-APC were obtained from Biolegend (San Diego, CA). Rapamycin (Rapa; LC laboratory, Rapa) was reconstituted in ethanol at 60 mg/mL and then diluted in the vehicle containing sodium CMC (C-5013 high viscosity, Sigma-Aldrich) and polysorbate 80. Rapa was administered intraperitoneally at a dose of 4 mg/kg every other day for 14 days. 0.2% CMC and 0.25% polysorbate 80 was administered as control solution.

### Quantitative real-time PCR assay

RNA was extracted with RNAeasy Mini kits (Omega) and was reversely transcribed into cDNA, real-time PCR was performed according to the manufacturer's instructions(Takara). The starting quantity of Tsc1and Tsc2 was normalized to the starting quantity of housekeeping gene Hprt for each sample. Primers were used in the present study as follows: TSC1 Sense: 5′-AAGCATCCTGACACCACCAAG-3′;

Reverse: 5′-GGATCTCCAGTTCCAACACCC-3′;

TSC2 Sense: 5′-CTTAGGTGGACTGGATGTATGTGG-3′; Reverse: 5′-TTGAACTGGCCCTTAATGGTG-3′

HPRT Sense: 5′-AGTACAGCCCCAAAATGGTTAAG-3′;

Reverse: 5′-CTTAGGCTTTGTATTTGGCTTTTC-3′.

### Immunofluorescence staining and flow cytometry (FCM)

Cells of peripheral LNs(pLNs), mesenteric LNs(mLNs), splenocytes and thymocytes were prepared and washed twice with ice-cold FACS buffer (PBS, pH 7.2, containing 0.1% NaN_3_ and 0.5% BSA). 5×10^5^ cells were resuspended in 100 µL FACS buffer and blocked by anti-mouse FcR mAb (2.4G2) followed by incubation with fluorochrome conjugated mAbs at 4°C for 30 minutes. Cells were washed twice with FACS buffer and at least ten thousand cells were assayed using a FACS Calibur (Becton Dickinson, CA) or Beckman Coulter Epics XL, and data were analyzed with CellQuest software.

### Isolation of liver lymphocytes

Four-to-5 week old mice were anesthetized and the liver was perfused with PBS containing heparin with a needle inserted into the portal vein. The liver was then removed and thoroughly dissected and passed through a 200-gauge stainless steel mesh, cells were suspended in RPMI 1640 medium containing 10% FBS and centrifuged. The pellet was resuspended in 40% Percoll solution containing 100 U/ml heparin, and then gently loaded on the layer of 70% Percoll solution followed by centrifugation at 2,000 rpm for 20 min at room temperature. The cells were collected from the Percoll interface and harvested by centrifugation and washed twice with FACS buffer.

### Adoptive transfer experiment

Naïve CD8^+^CD44^low^ T cells were purified from pLNs of CD45.1 (WT) or CD45.2(Tsc1 KO) mice by FACS™ Aria II. The purity and viability of enriched naïve CD8^+^T cells were approximately 98.5% or above. Cells were adoptively transferred into Rag1^−/−^ mouse recipients either separately or at 1∶1 ratio(3×10^5^ cells each). CD45.1 mice were sub-lethally irradiated(4 Gy) and transferred with either 6×10^5^ CD45.2(wild-type) or CD45.2(Tsc1 KO) naïve CD8^+^CD44^low^ T cells. Seven days after cell transfer, mice were sacrificed and peripheral lymphoid organs were collected, the percentage and absolute number of donor CD8^+^ T cells gated on CD45.1 or CD45.2 were analyzed and calculated.

### Naïve CD8^+^T cell culture

The sorted naïve CD8^+^CD44^low^ T cells were cultured in RPMI1640 supplemented with 10% FBS, 25 mM HEPES, 2 mM L-glutamine, 100 IU/mL of penicillin, 100 µg/mL streptomycin and 50 µM 2-mercaptoethanol (Sigma, St. Louis, MO, USA). Where indicated, IL-7 or IL-15 was supplemented into media at a concentration of 10 ng/mL (Miltenyi Biotech) and 50 ng/mL(PeproTech), respectively. Trypan blue exclusion assay and PI staining were used to determine the cell viability of sorted naïve CD8^+^CD44^low^ T cells at indicated time points.

### Western blot analysis

Cells were collected and wash twice with PBS and then lysed for 30 minutes on ice in RIPA solution containing protease inhibitor cocktails (Roche) together with phosphatase inhibitors (Sigma) as described previously. Equivalent protein concentrations were subjected to 8–12% SDS-PAGE (Bio-Rad, Hercules, CA). Rabbit anti–TSC1(#4906), rabbit anti–pS6(S235/236, #4858), rabbit anti–pS6(S240/244, #4838), rabbit anti-pAkt(S473, #4058), rabbit anti-pSTAT5(#4906), rabbit anti- pFoxO1/FoxO3a(#9464) and rabbit anti-Bim(#2933) were purchased from Cell Signaling Technology. Rabbit anti–TSC2(#1613-1) was purchased from Epitomics. Rabbit anti–Bcl-2(sc-7382, Santa Cruz Biotechnology, CA) or mouse anti-Beta-actin(Sigma-Aldrich). HRP labeled goat anti-rabbit IgG (Invitrogen, CA) and HRP labeled goat anti-mouse IgG were used. Contrast and brightness were adjusted uniformly for each image.

### Statistical analysis

All data are presented as the mean+SD. Student's unpaired *t* test for comparison of means was used to compare groups. A *P* value less than 0.05 was considered to be statistically significant.

## Supporting Information

Figure S1 **The normal percentage and cell number of CD8^+^ T cells in the livers of Tsc1 KO mice.** Lymphocytes isolated from livers of WT or Tsc1 KO mice were stained with anti-CD45 and CD8 mAbs (**A**). The percentage (**B**) and total cell number (C) of CD45^+^CD8^+^ T cells in livers of WT or Tsc1 KO mice were summarized. One representative of two independent experiments with identical results was shown. No significant difference between WT and Tsc1KO mice was observed (P>0.05).
(PPT)
