# Supplementary figures and images for: TSC1/2 Signaling Complex Is Essential for Peripheral Naïve CD8+ T Cell Survival and Homeostasis in Mice
Source: PLoS One. 2012 Feb 21;7(2):e30592. doi: 10.1371/journal.pone.0030592 (PMC3283604; doi:10.1371/journal.pone.0030592)

## Slide 1
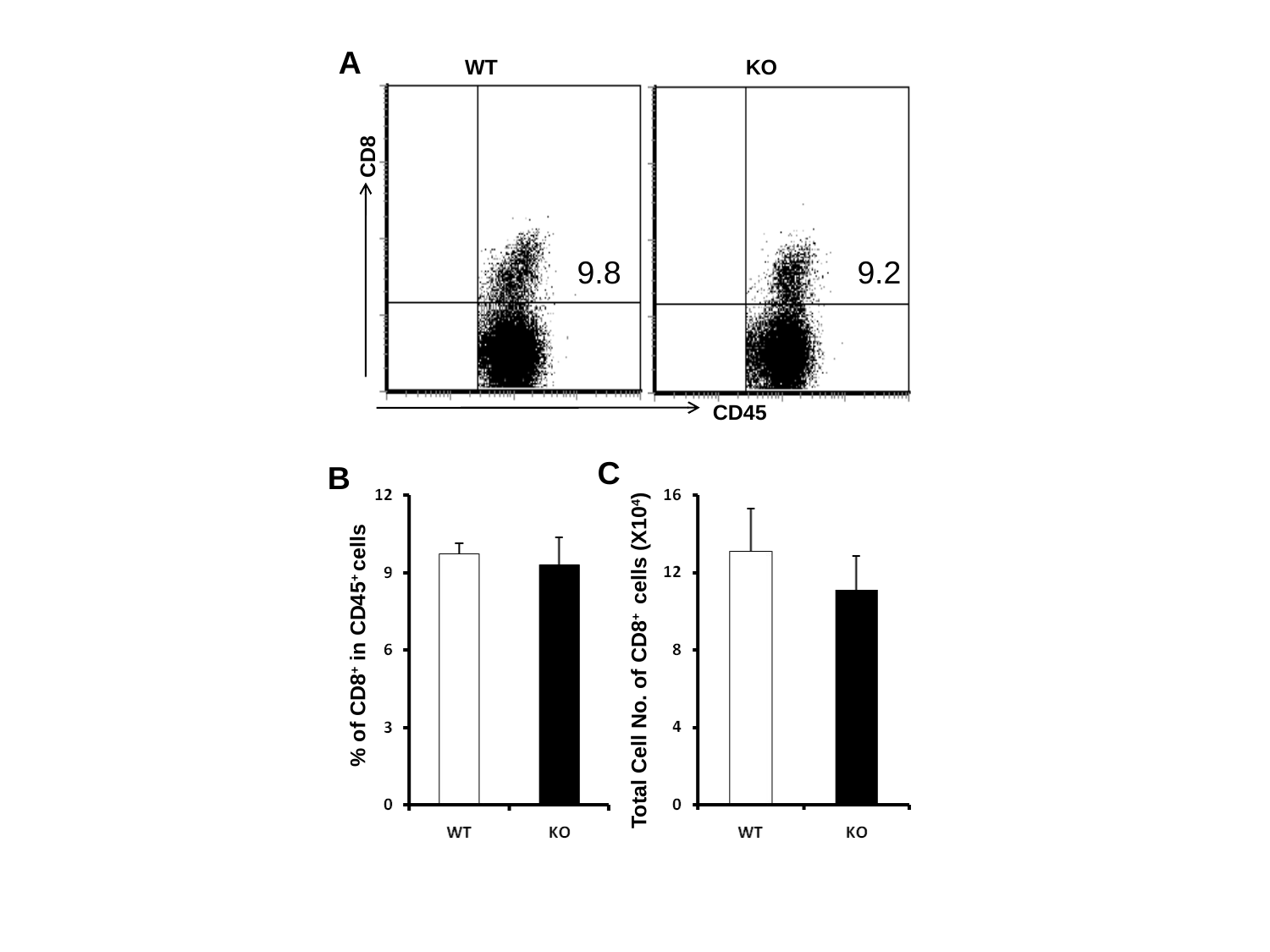

WT KO
CD8
9.8
9.2
CD45
A
B
% of CD8+ in CD45+ cells
Total Cell No. of CD8+ cells (X104)
C

Supplement: Figure S1 — The normal percentage and cell number of CD8+ T cells in the livers of Tsc1 KO mice. Lymphocytes isolated from livers of WT or Tsc1 KO mice were stained with anti-CD45 and CD8 mAbs (A). The percentage (B) and total cell number (C) of CD45+CD8+ T cells in livers of WT or Tsc1 KO mice were summarized. One representative of two independent experiments with identical results was shown. No significant difference between WT and Tsc1KO mice was observed (P>0.05). (PPT) [file pone.0030592.s001.ppt]
